# Particulate Air Pollution, Blood Mitochondrial DNA Copy Number, and Telomere Length in Mothers in the First Trimester of Pregnancy: Effects on Fetal Growth

**DOI:** 10.1155/2018/5162905

**Published:** 2018-11-05

**Authors:** S. Iodice, M. Hoxha, L. Ferrari, I. F. Carbone, C. Anceschi, M. Miragoli, A. C. Pesatori, N. Persico, V. Bollati

**Affiliations:** ^1^EPIGET LAB, Department of Clinical Sciences and Community Health, Università degli Studi di Milano, Milan, Italy; ^2^Department of Obstetrics and Gynecology ‘L. Mangiagalli', Fondazione IRCCS Ca' Granda Ospedale Maggiore Policlinico, Milan, Italy; ^3^Center of Excellence for Toxicological Research, Department of Medicine and Surgery, University of Parma, Italy; ^4^Department of Preventive Medicine, Fondazione IRCCS Ca' Granda Ospedale Maggiore Policlinico, Milan, Italy

## Abstract

Growing evidences have shown that particulate matter (PM) exposures during pregnancy are associated with impaired fetal development and adverse birth outcomes, possibly as a result of an exaggerated systemic oxidative stress and inflammation. Telomere length (TL) is strongly linked to biological age and is impacted by oxidative stress. We hypothesized that PM exposure during different time windows in the first trimester of pregnancy influences both mitochondrial DNA copy number (mtDNAcn), an established biomarker for oxidative stress, and TL. Maternal blood TL and mtDNAcn were analysed in 199 healthy pregnant women recruited at the 11th week of pregnancy by quantitative polymerase chain reaction. We also examined whether maternal mtDNAcn and TL were associated with fetal growth outcomes measured at the end of the first trimester of pregnancy (fetal heart rate, FHR; crown-rump length, CRL; and nuchal translucency, NT) and at delivery (birth weight, length, head circumference). The possible modifying effect of prepregnancy maternal body mass index was evaluated. PM_10_ exposure during the first pregnancy trimester was associated with an increased maternal mtDNAcn and a reduced TL. As regards ultrasound fetal outcomes, both FHR and CRL were positively associated with PM_2.5_, whereas the association with FHR was confirmed only when examining PM_10_ exposure. PM_10_ was also associated with a reduced birth weight. While no association was found between mtDNAcn and CRL, we found a negative relationship between mtDNAcn and fetal CRL only in overweight women, whereas normal-weight women exhibited a positive, albeit nonsignificant, association. As abnormalities of growth in utero have been associated with postnatal childhood and adulthood onset diseases and as PM is a widespread pollutant relevant to the large majority of the human population and obesity a rising risk factor, our results, if confirmed in a larger population, might represent an important contribution towards the development of more targeted public health strategies.

## 1. Introduction

Numerous health studies have shown the association between acute [1–5] and chronic [6–8] particulate matter (PM) exposures and the increase in mortality and morbidity risks in adults and children. In addition, growing evidences have shown that maternal exposure to PM during pregnancy might be associated with an impaired fetal development [9] and adverse birth outcomes [10], such as preterm birth and low birth weight [11] at term. The molecular mechanisms responsible for such effects are still mostly unclear, although studies have repeatedly evoked the role of oxidative stress and inflammation in mediating the effects of PM on human health [12].

Two of the main actors in the process of oxidative stress and inflammation are mitochondria and telomeres.

Mitochondria are cytoplasmic organelles which represent the major intracellular source and the preferred target of reactive oxygen species (ROS). Mitochondrial DNA copy number (mtDNAcn) correlates with the size and number of mitochondria within each cell [13] and is modulated by both endogenous and environmental factors [14]. PM exposure is a strong prooxidant stimulus that has been consistently associated with an mtDNAcn increase, as cells exposed to oxidative stress synthesize more copies of their mtDNA in order to compensate the damage. On the basis of these observations, alterations in mtDNAcn in various tissues, including whole blood, have emerged as a possible biomarker of mitochondrial dysfunction and risk factor for diverse cardiometabolic and neurodegenerative disorders as well as multiple cancers [15–17]. Notably, these diverse disorders have oxidative stress as a pathophysiological mechanism in common.

Increasing evidence that environmental exposure, such as smoking [18], benzene [19, 20], and ambient PM [21, 22], modifies mtDNAcn has begun to accumulate. Remarkably, a decreased placenta mtDNAcn was observed in relation to third trimester prenatal exposure to PM_10_ [23], and an altered cord blood mtDNAcn has been associated with adverse pregnancy outcomes, including an abnormal fetal growth [24].

Telomeres are located at the end of each chromosome and prevent DNA loss after each cell division in order to preserve the full genomic information [25]. Telomere length (TL) is strongly linked to biological age and is impacted by oxidative stress [26]. PM exposure has been associated with a modification in leukocyte TL, but this mainly concerns occupationally exposed subjects [27–29]; indeed, only limited evidence has been caused on pregnant women. Moreover, studies that have been conducted so far cover placenta or cord blood rather than maternal peripheral blood [30].

The aim of the present study is to determine the effects of exposure to PM_2.5_ and PM_10_ during the first trimester of pregnancy, on mtDNAcn and TL, in a sample of 199 healthy pregnant women recruited at the 11th week of pregnancy. We also evaluated the association among PM exposure, the abovementioned markers, and fetal growth parameters. Our hypothesis is that PM might increase maternal oxidative stress, accelerate telomere shortening, and finally impact on fetal growth.

## 2. Methods

### 2.1. Study Subjects

We recruited 199 healthy pregnant women at the “Clinica Mangiagalli”, Fondazione IRCCS Ca' Granda Ospedale Maggiore Policlinico, Milan, Italy, in the period between June 2014 and October 2015. Women aged 18 to 51 years with singleton pregnancies who were attending prenatal healthcare clinics in the 11th week of pregnancy were eligible for this study. Exclusion criteria include a history of illicit drug use, diabetes, hypertension, or some other chronic health conditions. A detailed informed consent form was signed by all participants, and the study was approved by the ethic committee of the Fondazione IRCCS Ca' Granda Ospedale Maggiore Policlinico. Information about demographics and lifestyle characteristics of the mother, such as smoking habits or alcohol consumption, was collected.

### 2.2. Fetal Ultrasound Measures and Birth Outcomes

Fetal measures were taken at the 11th week of pregnancy, as part of the prenatal screening test, when ultrasound fetal examination and drawing of blood (both for clinical assessment and for telomere/mtDNAcn measurements) were performed. During ultrasound examination, data about crown-rump length (CRL), nuchal translucency (NT), and fetal heart rate (FHR) were registered. Gestational age was calculated from the first day of the last menstrual period. At birth, we collected medical records of the newborns, obtaining data about gestational age at delivery, birth weight (BW), birth length (BL), and birth head circumference (BHC).

### 2.3. Exposure Assessment

Data on PM_10_ and PM_2.5_ were provided by Lombardy's Regional Environmental Protection Agency (ARPA) which regularly collects daily concentration of both pollutants using fixed monitoring stations of the Air Quality Monitoring Network. Daily exposure was calculated by averaging daily concentration of PM_2.5_ and PM_10_ from the available monitoring stations covering the city of Milan.

We assigned to each study subject twelve exposure cumulative intervals to pollutants obtained as cumulative mean of each gestational age week calculated from the last menstrual period date. The mean of gestational age week intervals ranges from the first week of pregnancy (0–1 w) to the entire first trimester (0–12 w). To account for missing data for a specific monitor, we used the information on the same pollutant and monitor on other days of the same year plus measurements of the same pollutant and day on the other available monitors [31].

### 2.4. Blood Collection and DNA Extraction

Blood was collected in EDTA tubes and processed within 2 hr of phlebotomy. EDTA-treated blood was centrifuged at 1200 × g for 15 min at room temperature to separate the buffy coat fraction from platelet-free blood plasma. The buffy coat fraction was transferred in a Cryovial and immediately frozen at −80°C until DNA extraction.

DNA was extracted using the Wizard Genomic DNA Purification Kit (Promega, Madison, WI, USA) following the manufacturer's instructions.

### 2.5. Telomere Length and mtDNAcn Measurement by Quantitative Real-Time PCR

TL and mtDNAcn were measured by using the real-time quantitative PCR method as described by Cawthon [32, 33] and Hou et al. [21].

These assays measure relative TL and relative mtDNAcn in DNA by determining, respectively, the ratio of telomere repeat copy number (T) and mitochondrial (mt) copy number to a single nuclear copy gene (S), which was the human (beta) globin (hbg). The T/S ratio and mt/S ratio are calculated in a given sample relatively to a reference pool DNA. The reference pool DNA was prepared from 50 DNA samples (1 *μ*g DNA for each sample).

A fresh standard curve prepared from the pooled DNA, ranging from 30 ng/*μ*l to 0.23 ng/*μ*l (serial dilutions 1 : 2), was included in every “T,” “mt,” and “S” PCR runs. For each sample, 9 ng of DNA was used as a template, and the reaction was run in triplicate. A high-precision MICROLAB STARlet Robot (Hamilton Life Science Robotics, Bonaduz AG, Switzerland) was used for transferring a volume of 7 *μ*l reaction mix and 3 *μ*l DNA (3 ng/*μ*l) in a 384-well format plate. All PCRs were performed on a 7900HT Fast Real-Time PCR System (Applied Biosystems). Primers were previously reported [21, 32, 33]. At the end of each real-time PCR reaction, a melting curve was added in order to confirm the amplification specificity and the absence of primer dimers. The average of the three T and three mt measurements was divided by the average of the three S measurements to, respectively, calculate the T/S or the mt/S ratio for each sample.

### 2.6. Statistical Analysis

Summary statistics for mother and newborn characteristics are presented as mean ± SD or frequency and percentage. The correlation between mtDNAcn and TL was examined. To investigate whether PM exposure was associated with mtDNAcn or TL, we evaluated the associations between daily PM concentrations in the first trimester of pregnancy (as gestational age week intervals) and mtDNAcn or TL. A univariate exploratory analysis was performed to select potential covariates associated with each outcome (i.e., age, sex, BMI, smoking habits, ethnicity, ovulation induction, parity, previous miscarriage, drug assumption before and during pregnancy, seasonal infections measured by the number of seasonal flu cases in Lombardy region (https://www.cirinet.it/jm/sorveglianza-virologica/stagioni-precedenti/clinico-epidemiologica.html), season, day and week of enrolment, humidity, temperature, and apparent temperature). The selection of the most appropriate model structures was based on the minimization of the Akaike information criterion (AIC). The best model selected was adjusted for age, smoking habits (never, past, or current smokers), season, maternal age, BMI (<25 kg/m^2^, BMI ≥ 25 kg/m^2^), and gestational week at examination. Dependent variables were log-transformed to achieve normality of models' residuals. Estimated effects are reported as geometric mean ratio (GMR) and 95% confidence intervals (CI) associated with an increase of 10 *μ*g/m^3^ in each pollutant. We used separate models to estimate the effects of PM_2.5_ and PM_10_ on each outcome.

We subsequently evaluated the association between mtDNAcn/TL and both fetal growth measures (FHR and CRL). All models were adjusted for the above-mentioned selected covariates.

We performed causal mediation analysis to identify potential pathways that could explain the observed associations between PM exposure and fetal growth. This approach examines how a third intermediate variable, i.e., the mediator, is related to the observed exposure-outcome relationship. In our study, we studied the potential mediator role of mtDNAcn in the association between PM and FHR (Supplementary Figure 2). For this analysis, we selected PM_10_ of the mean of the first 5 weeks of gestation (PM_10, 0-5w_) as it was the exposure associated with both FHR and mtDNAcn. To apply mediation models, three criteria must be satisfied. First, there must be a statistically significant association between exposure (PM_10_) and outcome (FHR). Second, the exposure (PM_10_) must have an effect on mediator (mtDNAcn), and third, the mediator must be associated with the outcome (FHR) when exposure is controlled (after adjusting for mtDNAcn). Unfortunately, in our study, the latter condition was not fulfilled; accordingly, we could not calculate a significant indirect effect (Supplementary Table 1).

In addition, we investigated (i) the association between PM and crown-rump length (CRL) by a multivariable regression model adjusted for smoking habits (never, past, or current smokers), season (winter, spring, summer, and autumn), maternal age, categorical BMI (<25 kg/m^2^, BMI ≥ 25 kg/m^2^), gestational week at examination, mtDNAcn, and interaction between categorical BMI and mtDNAcn and (ii) the association between PM and fetal hearth rate (FHR) by a multivariable regression model adjusted for smoking habits, season, maternal age, categorical BMI, gestational week at examination, TL, and interaction between categorical BMI and TL. Complete models were graphically explored only for the models showing the larger effects of PM on each fetal outcome. Due to a high number of comparisons, we took into account a correction for multiple comparisons based on the false discovery rate (FDR) control. A threshold of 0.05 was applied on FDR *P* value significance to identify the associations that remain significant after the correction. A two-tailed value of *P* < 0.05 was considered statistically significant. All statistical analyses were performed with SAS software version 9.4. Mediation analysis was executed while utilizing the PROCESS program (model 4) provided by Hayes (2013).

## 3. Results

Table 1 shows the characteristics of the study population. Most women were nonsmokers, with a mean age of 33 years and an average prepregnancy BMI of 22.5 kg/cm^2^. A total of 190 pregnancies ended in live births: 7 miscarriages were recorded and two mothers were lost to follow-up. Table 1 also reports newborn characteristics of the 190 live births, including ultrasound measurements at recruitment and size at birth.

As shown in supplementary Figure 1, the mean levels of ambient PM_10_ and PM_2.5_ measured in Milan the days before the examinations (0–12-week means) ranged from 10 to 90 *μ*g/m^3^ and from 7 to 69 *μ*g/m^3^, respectively.

**Figure 1:**
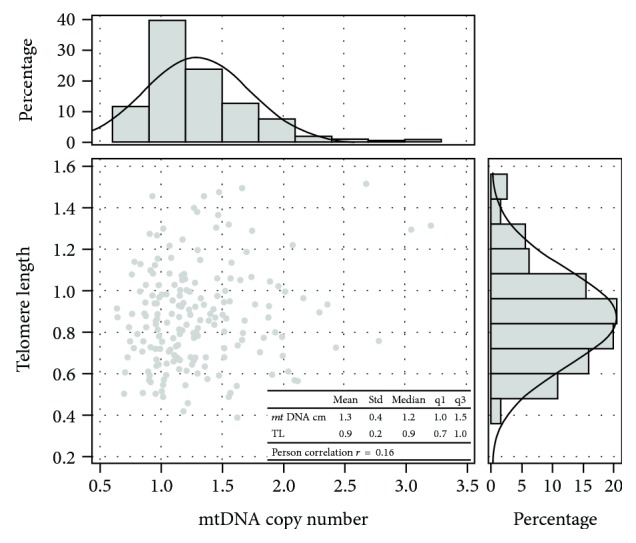
Correlation between TL and mtDNAcn.

We examined TL and mtDNAcn in DNA extracted from maternal whole blood sample of study subjects; as expected, age was inversely related to TL, even if not significantly (*β* = −0.01, *P* value > 0.10), but not related to mtDNAcn (*β* = 0.01, *P* value = 0.28). Smoking and maternal BMI did not show any association with the two markers measured. As reported in Figure 1, we observed a modest correlation between TL and mtDNAcn (Pearson correlation coefficient: 0.16).

**Figure 2:**
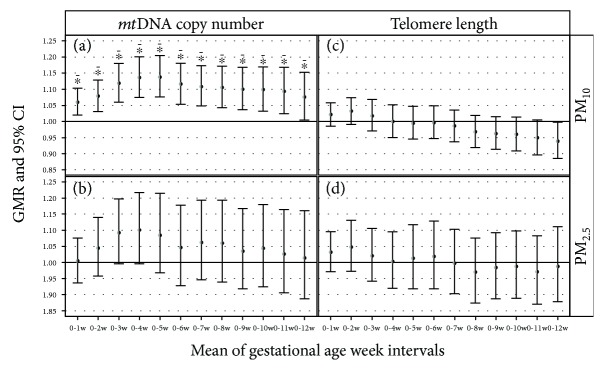
Association of *mt*DNA copy number and telomere length with PM at the mean of gestational age week intervals. Geometric mean ratio (GMR) of *mt*DNAcn and TL for an increase of 10 *μ*g/m^3^ of PM was adjusted for age, categorical BMI (<25 kg/m^2^, BMI ≥ 25 kg/m^2^), smoking habits (never, past, or current smokers), season, and gestational week at examination. The asterisk indicates a significant association; the dotted asterisk indicates a statistically significant association with a pFDR ≤ 0.05.

Considering the association between PM exposure and mtDNAcn, we observed in all the gestational age intervals examined a PM-related increase in mtDNAcn for PM_10_ exposure, after adjusting for age, BMI, smoking habit, season, and gestational age at the examination (Figure 2(a)). The effect was maximum for the average of the first 5 weeks of pregnancy (adjusted GMR = 1.14; 95% CI: 1.08, 1.20; *P* < 0.001). An increase was also reported for PM_2.5_ exposures even though the associations were not statistically significant (Figure 2(b)).

On the contrary, we observed a negative and significant effect of the 12-week mean PM_10_ on TL (adjusted GMR = 0.94; 95% CI: 0.88, 0.99; *P* = 0.038) (Figure 2(c)). The effect was not observed for PM_2.5_ exposure (Figure 2(d)).

**Figure 3:**
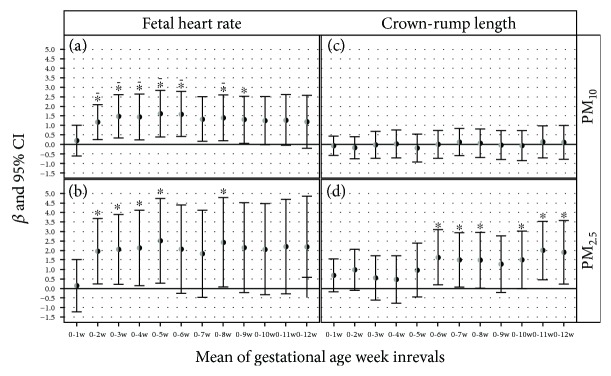
Effect of PM on FHR and CRL measured at the 11th week of pregnancy. FHR model adjusted for smoking habits (never, past, or current smokers), season, age, categorical BMI (<25 kg/m^2^, BMI ≥ 25 kg/m^2^), gestational week at examination, TL, and interaction between categorical BMI and TL; CRL model adjusted for smoking habits, season, age, categorical BMI, gestational week at examination, *mt*DNAcn, and interaction between categorical BMI and *mt*DNAcn. Each panel reported *β* coefficients and 95% CI for PM_10_ (a) and PM_2.5_ (b) on fetal heart rate and for PM_10_ (c) and PM_2.5_ (d) on crown-rump length calculated at each week interval. *β*s are calculated for a 10 *μ*g/m^3^ increase in PM_10_ and PM_2.5_. The asterisk indicates a significant association; the dotted asterisk indicates a statistically significant association with a pFDR ≤ 0.05.

We further investigated the possible association between PM exposure and fetal outcomes (i.e., fetal heart rate, crown-lump length, and nuchal translucency) measured by ultrasound at the time of enrolment. PM_10_ was associated with an increased FHR from the gestational age week interval 0–2 (Figure 3(a)), ranging from an adjusted estimate of 1.16 in weeks 0–2 to an adjusted estimate of 1.61 in weeks 0–5. A similar trend was observed for PM_2.5_ exposure (Figure 3(b)). In addition, CRL was positively associated only with PM_2.5_ exposure (Figures 3(c) and 3(d)).

We investigated whether mtDNAcn could represent a mediator of the association between PM and FHR (Supplementary Figure 2). However, as we tried to formally investigate this possibility, we found that our data did not fulfill the necessary and sufficient conditions to establish mediation (Supplementary Table 1). This finding let us infer that mtDNAcn is not the mediator of the association.

**Figure 4:**
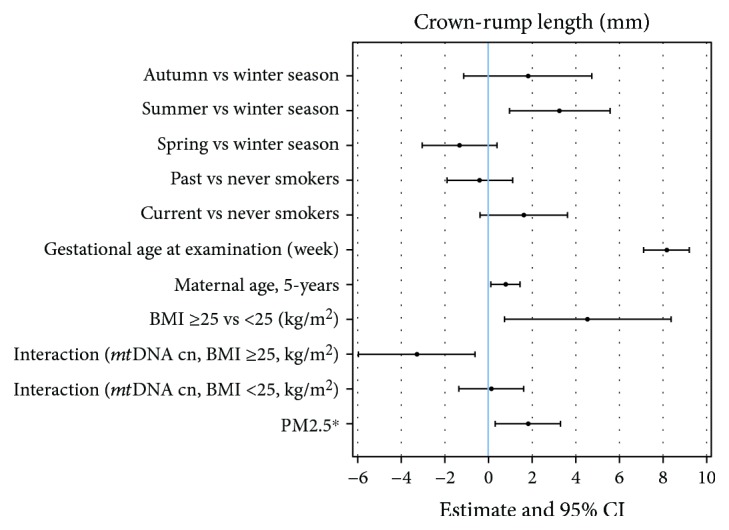
Complete CRL model showing the magnitude of association of each variable entered in the multivariable linear regression model as a covariate. *P* value of interaction between BMI and *mt*DNAcn was <0.001. ^∗^The effect on CRL was evaluated for each 10 *μ*g/m^3^ increase in PM_2.5_ of the mean of the first 11 weeks of gestation.

No association was found between mtDNAcn and fetal outcomes (Supplementary Table 2). Nevertheless, when the interaction between mtDNA and CRL was taken into account, we observed a strong modifying effect of maternal BMI in modulating association between mtDNAcn and CRL. In Figure 4, we reported the beta estimates of each variable included as covariate in the multivariable linear regression model investigating the association between mtDNAcn and CRL. An interaction test formally performed to assess effect modification between BMI and mtDNAcn was statistically significant (*P* value < 0.001), indicating a sensibly larger association among overweight subjects. Interestingly, in women with a prepregnancy BMI above 25 (overweight), we observed a clear inverse relationship between mtDNAcn and CRL, whereas in normal-weight women, mtDNAcn was not associated with CRL.

When we applied the same approach to investigate the determinants of FHR and possible interactions among variables (Supplementary Figure 3), the association of the TL with FHR was not significant, and the interaction test performed to assess effect modification between BMI and TL was not statistically significant (*P* value = 0.106). However, a diverse association was observed in normal-weight (positive association) and overweight (negative association) women.

No associations were observed either with TL and mtDNAcn or with nuchal translucency (NT) (data not shown).

**Figure 5:**
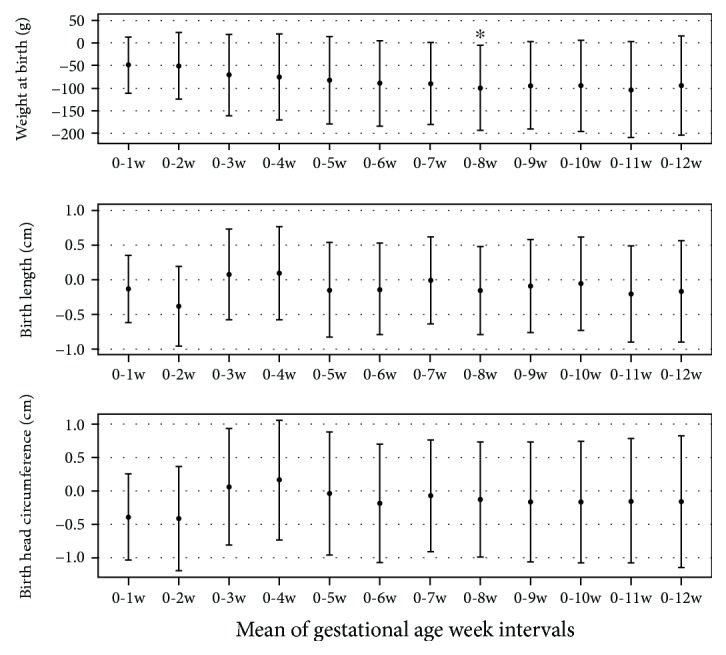
Association of birth outcome with PM_10_ at the mean of gestational age week intervals. Estimate of birth weight, birth length, and birth head circumference for an increase in 10 *μ*g/m^3^ of PM was adjusted for maternal age, BMI, smoking habits, season, gestational week at examination, *mt*DNAcn, and TL.

The analysis of the association with the first trimester PM_10_ exposure on birth weight, applying a model adjusted for maternal age, smoke, season, maternal BMI, gestational age, mtDNAcn, and TL, showed a negative association for different time windows that reached significance at weeks 0–8 (adjusted estimate = −0.99; 95% CI: −194, −3.88; *P* = 0.042). Exposure was instead not associated with birth length and birth head circumference (Figure 5). No associations resulted between PM_2.5_ exposure and any of the outcomes considered (data not shown).

## 4. Discussion

The main goal of the present study was to examine whether exposure to particulate matter (PM_10_ and PM_2.5_) experienced from the mother in the first trimester of pregnancy was associated with oxidative stress (estimated as mtDNAcn) and maternal TL. We also examined whether maternal mtDNAcn and TL were associated with fetal growth outcomes measured at the end of the first trimester of pregnancy (FHR, CRL, and NT) and at delivery (birth weight, length, and head circumference). The possible modifying effect of prepregnancy maternal BMI was evaluated.

PM_10_ exposure, considered in different week windows of the first pregnancy trimester, was associated with an increased maternal mtDNAcn and a reduced TL. As regards ultrasound fetal outcomes, both FHR and CRL were positively associated with PM_2.5_, whereas only the association with FHR was confirmed when examining PM_10_ exposure. PM_10_ was also associated with a reduced birth weight.

Our findings let us infer that mtDNAcn is not the mediator of the association between PM and FHR as our data did not fulfill the necessary and sufficient conditions to establish mediation. We therefore speculate that probably two independent pathways linked to PM exposure exist.

In addition, when we examined the possible modifying role of BMI, we found a negative relationship between mtDNAcn and fetal CRL only in overweight women, whereas normal-weight women exhibited a positive, albeit nonsignificant, association.

Pregnancy is a physiological condition characterized by an increased susceptibility to oxidative stress and inflammation. Placenta, in particular, has a central role in this context, as the high placental mitochondrial activity gives rise to an increased ROS production [34]. Ideally, this increased concentration of ROS should be balanced by an increase in antioxidant compounds and enzymes [35]. Increased mtDNAcn has been associated with adverse pregnancy outcomes, such as low and high birth weight [24], placental abruption [36], and preeclampsia [37].

PM exposure has been previously linked to an increased oxidative stress in occupational [21] and general environment settings [22, 38] as well as to a modification in TL [27, 29, 39]. In pregnant women, PM exposure has been also associated with a modification of oxidative stress measured in the placenta and in the cord blood at delivery [30, 40]. Most of the previous investigations examined placental mtDNAcn in relationship to PM exposure experienced during the last period of pregnancy and showed increased oxidative stress and TL shortening [23].

To the best of our knowledge, this is the first investigation focusing on oxidative stress measured in maternal blood collected at the end of the first trimester of pregnancy. Our findings confirm the relationship between PM exposure and both increased mtDNAcn as marker of oxidative stress and TL shortening. Interestingly, examining the possible correlation between TL and mtDNAcn, we found a correlation coefficient very similar to the ones previously reported in different experimental settings, such as elderly women [41] and in a female subgroup of the EPIC cohort [42].

Quite surprisingly, fetuses, whose mothers had an increased exposure, had a faster heart rate and higher CRL (measured at the 11th week of pregnancy), whereas in literature, PM exposure has been mostly related to smaller fetuses measured in more advanced stages of pregnancy. One possible explanation of this unexpected result could be speculative: at an early gestational age PM exposure might act with a selection mechanism that lead only stronger fetuses to survive.

Although alterations of fetal heart rate have been considered a symptom of fetal distress, the very large majority of studies investigated FHR at the third trimester of pregnancy. Thus, the meaning of the association we found between PM exposure and FHR is difficult to interpret and should be further investigated.

The negative effect of PM on weight at birth is instead coherent with current literature [43–45].

An additional comment regards the general lack of association with PM_2.5_, whereas we observe an effect associated with PM_10_ exposure. This finding is somehow surprising but it must be interpreted taking into account two factors. First, PM_10_ dataset available for the Lombardy region in the study period was more complete, and it was characterized by a better spatial resolution; thus, this allows a better estimate of exposure levels. Second, in the study area, PM_10_ is mainly constituted by fine particles, and PM_2.5_ represents 58–94% of PM_10_ [46].

The present study must be interpreted taking into account both strength and limitations. First, although the study population is not very large compared to other studies conducted on pregnant women, we collected very detailed information about possible confounding factors, and we were able to consider them in statistical analyses. Moreover, all study participants were recruited in the same hospital, and ultrasound measurements were performed by gynecologists after a standardized training program, using the same instrumentation.

A limitation of the study is given by the possible inaccuracy in estimating gestational age. Gestational age was calculated from the first day of the last menstrual period, and, indeed, this measurement is affected by menstrual irregularities. However, the possible measurement error is supposed to be randomly distributed, thus not affecting the relationship with PM exposure. On the contrary, the possible use of ultrasound measures to correct gestational age might have led to a systematic error due to the possible effect of PM exposure on fetal size. Finally, fetal ultrasound has a great deal of measurement error, and therefore, measurement error might drive the observed results, although such error should cause nondifferential exposure misclassification, and it seems unlikely to be driving the results.

The inclusion criteria of this study were very strict, as we enrolled only healthy women, with no comorbidities.

This choice has the advantage of examining a homogenous population and of limiting any possible confounders, although it prevents from the evaluation on particularly susceptibility conditions such as diabetes or hypertension.

Although the sample size of our study is limited, we were able to assess significant associations between PM and fetal growth and between PM and mtDNAcn/LT; however, we were not able to confirm a mediation role of mtDNAcn. Further studies performed on a larger population might help to elucidate the link between PM, mtDNAcn/LT, and fetal growth. In conclusion, in the present work, PM exposure was associated with an increased oxidative stress and a reduced TL measured in maternal blood at the end of the first trimester of pregnancy. The PM exposure experienced during the first trimester was also associated with CRL, FHR, and birth weight. As abnormalities of growth in utero has been associated with postnatal childhood and adulthood onset diseases, since PM is a widespread pollutant relevant to the large majority of the human population and obesity is a rising risk factor, our results, if confirmed in a larger population, might represent an important contribution towards the development of more targeted public health strategies.

## Figures and Tables

**Table 1:** Description of study population.

	Mean ± SD or range and number
*Mother* (*n* = 199)	
Age (years)	33.0 (3.9)
Gestational age at examination (weeks)	11.9 (0.5)
Prepregnancy BMI (kg/m^2^)	22.5 (4.0)
BMI, categorical	
BMI < 25 kg/m^2^	155 (77.9)
BMI ≥ 25 kg/m^2^	44 (22.1)
Self-reported smoking status	
Never smoker	151 (75.9)
Past smoker	30 (15.1)
Current smoker	18 (9.1)
Parity	
0	121 (60.8)
1	67 (33.7)
2	10 (5.0)
3	1 (0.5)
Season of enrolment	
Autumn	63 (31.7)
Winter	41 (20.6)
Spring	41 (20.6)
Summer	54 (27.1)

*Newborn* (*n* = 190^∗^)	
Sex	
Male	108 (56.8)
Female	82 (43.2)
Gestational age at delivery (weeks)	38.8 (1.4)
Birth weight (g)	3272.9 (477.9)
Birth length (cm)	49.9 (2.0)
Birth head circumference (cm)	34.2 (1.5)
Crown-rump length (CRL) (mm)	62.2 (6.9)
Nuchal translucency (NT) (cm)	1.9 (0.4)
Fetal heart rate (FHR) (bpm)	160.4 (6.1)

^∗^7 miscarriages and two mothers lost to follow-up.

## Data Availability

The data used to support the findings of this study are available from the corresponding author upon request.

## Supplementary Materials

Supplementary Materials Supplementary Figure 1: box plot showing the distribution of PM_10_ and PM_2.5_ concentrations for the exposure intervals defined as the mean of gestational age weeks. Supplementary Figure 2: conceptual and statistical diagram of mediation analysis. Supplementary Table 1: mediation analysis. Supplementary Table 2: linear regression model with crown-rump length and fetal heart rate as dependent variables and mtDNAcn and telomere length as independent variables. Supplementary Figure 3: complete FHR model showing the magnitude of effect of each variable entered in the multivariable linear regression model as a covariate. The *P* value of interaction between BMI and TL was 0.106.
