# Supplementary material for: Particulate Air Pollution, Blood Mitochondrial DNA Copy Number, and Telomere Length in Mothers in the First Trimester of Pregnancy: Effects on Fetal Growth
Source: Oxid Med Cell Longev. 2018 Nov 5;2018:5162905. doi: 10.1155/2018/5162905 (PMC6247572; doi:10.1155/2018/5162905)
Supplement: Supplementary Materials — Supplementary Figure 1: box plot showing the distribution of PM10 and PM2.5 concentrations for the exposure intervals defined as the mean of gestational age weeks. Supplementary Figure 2: conceptual and statistical diagram of mediation analysis. Supplementary Table 1: mediation analysis. Supplementary Table 2: linear regression model with crown-rump length and fetal heart rate as dependent variables and mtDNAcn and telomere length as independent variables. Supplementary Figure 3: complete FHR model showing the magnitude of effect of each variable entered in the multivariable linear regression model as a covariate. The P value of interaction between BMI and TL was 0.106. [file 5162905.f1.docx]

Supplementary material

**Particulate air pollution, blood mitochondrial DNA copy number and telomere length in mothers in the first trimester of pregnancy: effects on fetal growth**

Iodice S.^1^, Hoxha M. ^1^, Ferrari L^1^., Carbone I.F.^2^, Anceschi C. ^1^, Miragoli M. ^3^, Pesatori AC^1,4^, Persico N. ^2^, Bollati V.^1,4^

1 EPIGET LAB, Department of Clinical Sciences and Community Health, Università degli Studi di Milano, Milan, Italy.

2 Department of Obstetrics and Gynecology 'L. Mangiagalli', Fondazione IRCCS Ca’ Granda Ospedale Maggiore Policlinico, Milan, Italy.

3 Center of Excellence for Toxicological Research, Department of Medicine and Surgery, University of Parma, IT.

4 Department of Preventive Medicine, Fondazione IRCCS Ca’ Granda Ospedale Maggiore Policlinico, Milan, Italy.


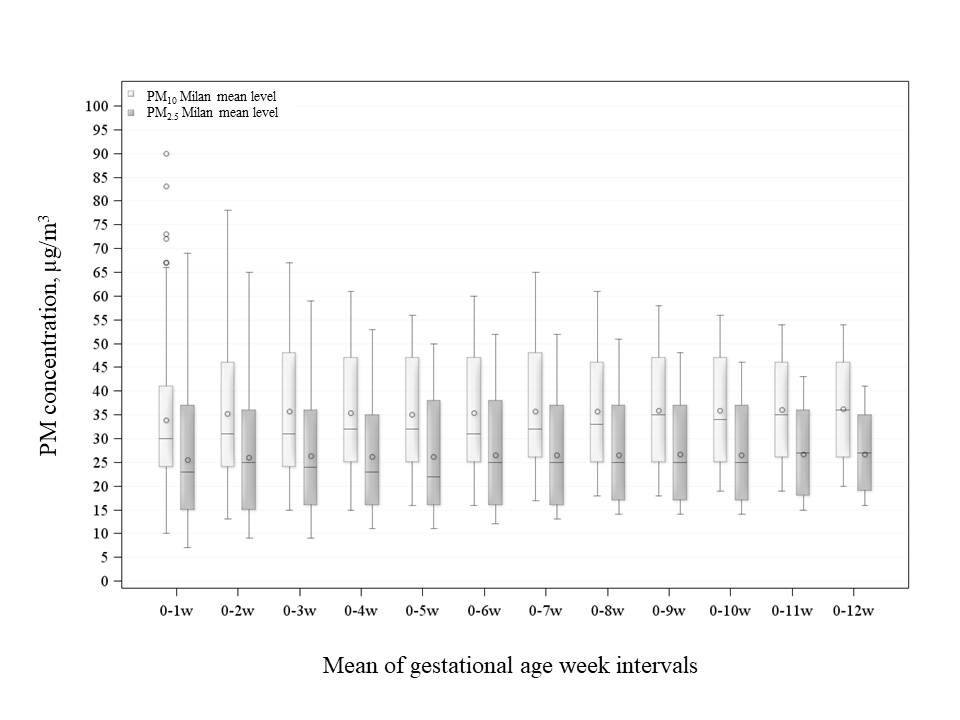


**Supplementary figure 1** Box plot showing the distribution of PM_10_ and PM_2.5_ concentrations for the exposure intervals defined as mean of gestational age weeks.

Supplementary figure 2: Conceptual and statistical diagram of mediation analysis.


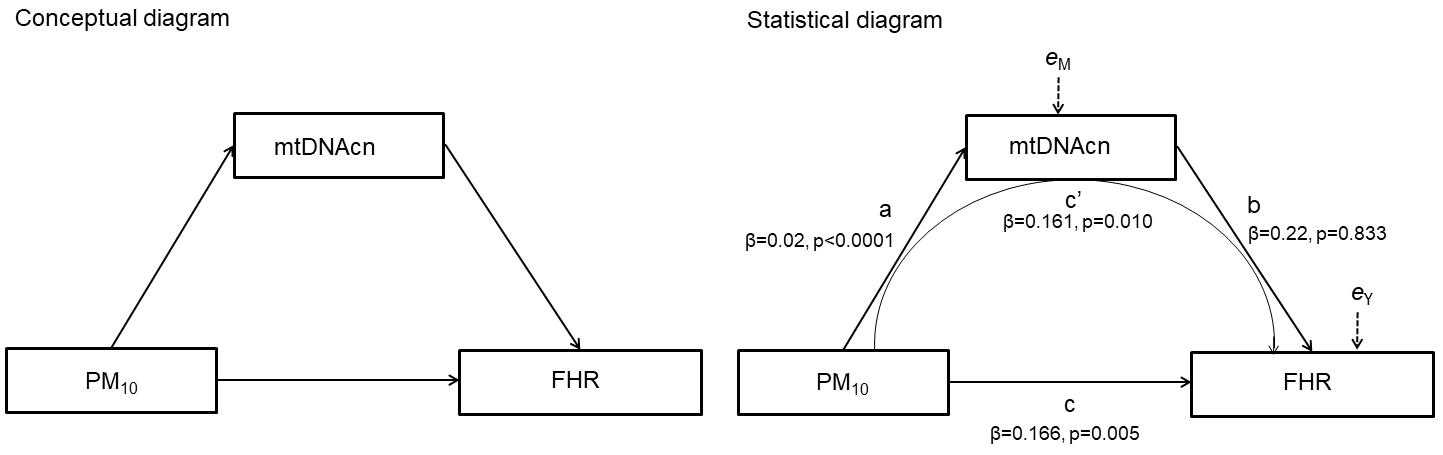


In the conceptual diagram PM_10_ is the independent variable, *mt*DNA cn is the possible mediator and FHR is the dependent variable. All models were adjusted for age, categorical BMI (<25 kg/m², BMI≥25 kg/m²), smoking habits (never, past or current-smokers), season and gestational week at examination. Beta were reported for an increase in 10 µg/m^3^ of the mean of first 5 weeks of gestation and for 1 unit increase in mtDNAcn. The statistical diagram shows the estimated linear regression coefficients.

Supplemetary table 1. Mediation analysis

| **Predictors** | **Y variable** | **Path** | **Beta** | **SE** | **P-value** |
| --- | --- | --- | --- | --- | --- |
| *Model1* |  |  |  |  |  |
| PM_10, 0-5w_ | *mt*DNAcn | a | 0.180 | 0.041 | <.0001 |
| *Model 2* |  |  |  |  |  |
| PM_10, 0-5w_ | FHR | c (total effect) | 1.6537 | 0.5848 | 0.005 |
| *Model 3* |  |  |  |  |  |
| PM_10, 0-5w_ | FHR | c' (direct effect) | 1.614 | 0.616 | 0.010 |
| *mt*DNA cn |  | b | 0.222 | 1.051 | 0.833 |
|  |  |  | | | |
|  |  | **Indirect effect of PM on FHR (path a x b)** | | | |
|  |  | Beta | boot SE | boot low LLCI | boot ULCI |
| Bootstrapping |  | 0.040 | 0.230 | -0.460 | 0.478 |
|  |  | Beta | SE | P-value |  |
| Normal theory |  | 0.040 | 0.195 | 0.837 |  |

All models were adjusted for age, categorical BMI (<25 kg/m², BMI≥25 kg/m²), smoking habits (never, past or current-smokers), season and gestational week at examination. Beta were reported for an increase in 10 µg/m^3^ of the mean of first 5 weeks of gestation and for 1 unit increase in mtDNAcn.

SE: standard error, Boot: bootstrapped , LLCI: lower limit confidence interval, ULCI: upper limit confidence interval.

Supplementary table 2. Linear regression model with crown-rump length and fetal heart rate as dependent variable and mtDNAcn and telomere length as independent variable

|  | **independent variable** | **beta (95% CI)** | **P-value** |
| --- | --- | --- | --- |
| **Crown-rump length** | |  |  |
|  | ***mt*DNAcn** | -0.94 (-2.19, 0.31) | 0.139 |
|  | **Telomere length** | -0.93 (-3.37, 1.52) | 0.456 |
| **Fetal heart rate** |  |  |  |
|  | ***mt*DNAcn** | 1.07 (-0.94, 3.07) | 0.295 |
|  | **Telomere length** | 0.27 (-3.65, 4.19) | 0.892 |

Models were adjusted for smoking habits (never, past or current-smokers), season, age, categorical BMI (<25 kg/m², BMI≥25 kg/m²), gestational week at examination.


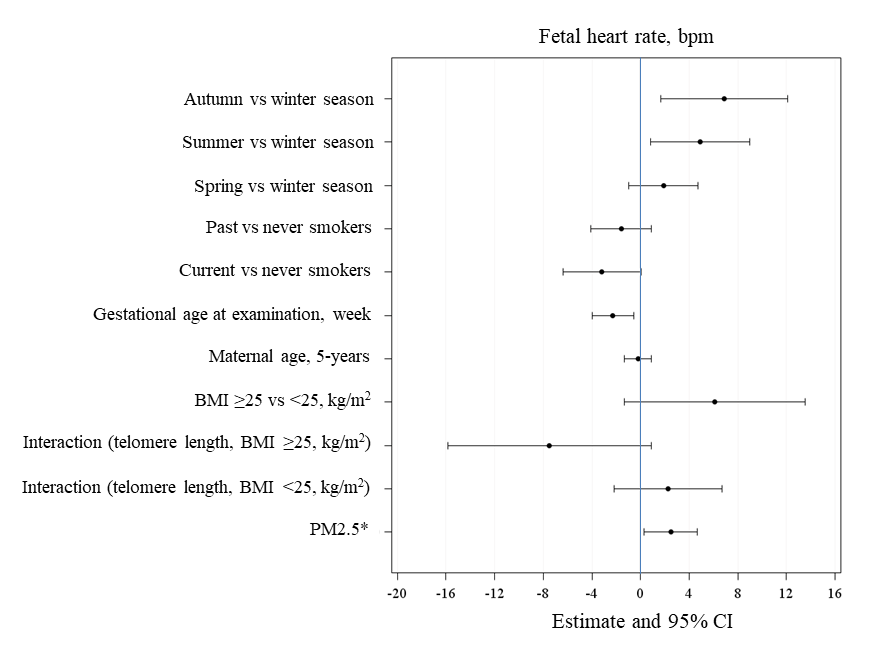


**Supplementary figure 3** Complete FHR model showing the magnitude of effect of each variable entered in the multivariable linear regression model as covariate. P-value of interaction between BMI and TL was = 0.106.

*The effect on FHR was evaluated for each 10 μg/m^3^ increase in PM_2.5_ of the mean of first 5 weeks of gestation.
